# Clinical applications of digital angiography with the harmonization function in body interventional radiology

**DOI:** 10.1007/s11604-020-00990-w

**Published:** 2020-05-19

**Authors:** Hidekatsu Tateishi, Kazunori Kuroki, Haruhiko Machida, Toshihiko Iwamoto, Toshiya Kariyasu, Yuusuke Kinoshita, Masanaka Watanabe, Hisae Shiga, Saori Yuda, Kenichi Yokoyama

**Affiliations:** 1 Department of Radiology, Kyorin University Faculty of Medicine, 6-20-2 Shinkawa, Mitaka, Tokyo 181-8611 Japan; 2 Section of Radiology, Kyorin University Hospital, 6-20-2 Shinkawa, Mitaka, Tokyo 181-8611 Japan

**Keywords:** Digital angiography, Dynamic density optimization, Harmonization function, Interventional radiology, Radiation exposure

## Abstract

Digital subtraction angiography (DSA) is frequently applied in interventional radiology (IR). When DSA is not useful due to misregistration, digital angiography (DA) as an alternative option is used. In DA, the harmonization function (HF) works in real time by harmonizing the distribution of gray steps or reducing the dynamic range; thus, it can compress image gradations, decrease image contrast, and suppress halation artifacts. DA with HF as a good alternative to DSA is clinically advantageous in body IR for generating DSA-like images and simultaneously reducing various motion artifacts and misregistrations caused by patient body motion, poor breath-holding, bowel and ureter peristalsis, and cardiac pulsation as well as halation artifacts often stemming from the lung field. Free-breath DA with HF can improve body IR workflow and decrease the procedure time by reducing the risk of catheter dislocation and using background structures as anatomical landmarks, demonstrating reduced radiation exposure relative to DSA. Thus, HF should be more widely and effectively utilized for appropriate purposes in body IR. This article illustrates the basic facts and principles of HF in DA, and demonstrates clinical advantages and limitations of this function in body IR.

**Electronic supplementary material:**

The online version of this article (10.1007/s11604-020-00990-w) contains supplementary material, which is available to authorized users.

## Introduction

Digital subtraction angiography (DSA) is a fluoroscopic technique used frequently in interventional radiology (IR) for visualizing blood vessels [1–6]. DSA eliminates radiopaque structures such as bones by digitally subtracting the pre-contrast mask image from angiographic images acquired following injection of contrast media into vessels via the catheter and offers critical information particularly by improving the delineation of small and peripheral vessels. As a major problem in DSA, misregistration occurs when the mask and angiographic images do not exactly coincide [1, 2]. It is caused not only by voluntary movement of the patient but also by involuntary movements such as bowel peristalsis or cardiac pulsation. When image quality and interpretability are severely degraded due to the misregistration, IR procedures should be performed by referring to digital angiography (DA) images without subtraction. In regions overlapped by radiopaque structures such as bones, however, the successful conduct of detailed assessments using DA images is limited. In DA, halation artifacts also occur in regions with a great X-ray attenuation difference. To overcome these shortcomings of both DSA and DA, a harmonization function (HF) has been made clinically applicable to offer DSA-like images in DA with a flat-panel detector (FPD) system.

In DA images, this image processing function works in real time by harmonizing the distribution of gray steps or reducing the dynamic range; thus, it can compress image gradations, decrease image contrast, and suppress halation artifacts [7–9]. HF has been used frequently during angiography or IR of the lower extremities where misregistration secondary to patient body movement can easily occur in DSA images specifically due to pain and/or a heat sensation with the injection of contrast media. HF has been made commercially available as the dynamic density optimization (DDO) function or “CLEAR LEG” from Siemens Healthineers AG (Erlangen, Germany) [7, 8]. To our knowledge, the clinical application of HF in body IR has never before been described. The purpose of this review article was, thus, to illustrate basic facts and principles of HF in DA and demonstrate clinical advantages and limitations of this function in body IR.

## Basic facts and principles

**Fig. 1:**
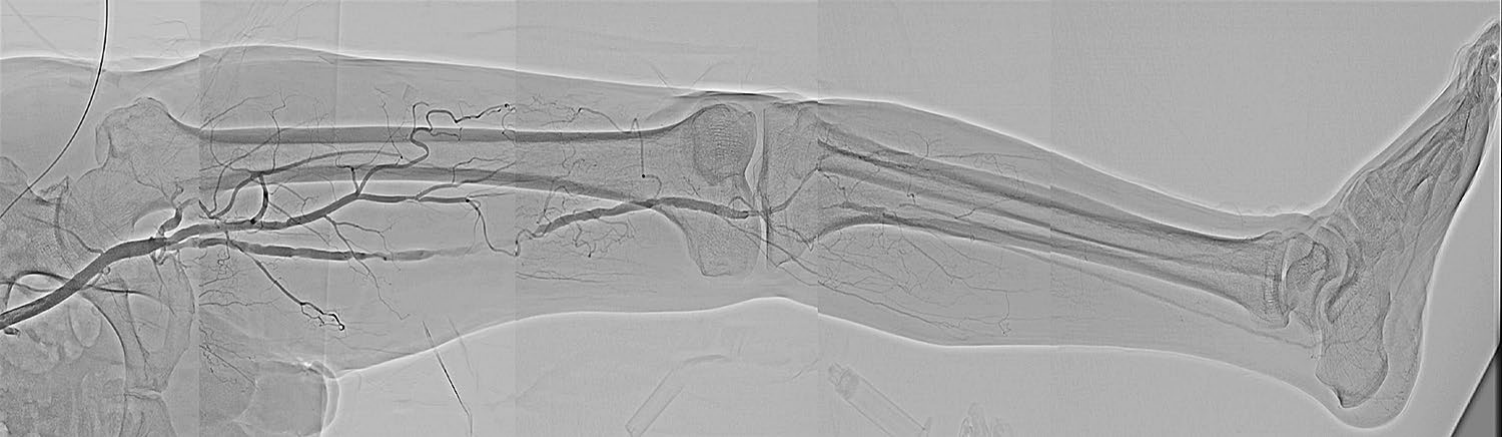
Stepping DA of the lower extremity with HF in an 84-year-old male with peripheral arterial disease. Stepping DA with HF following a single injection of contrast media well delineates the arteries of the whole lower extremity with less of an overlapped background relative to without HF

**Fig. 2:**
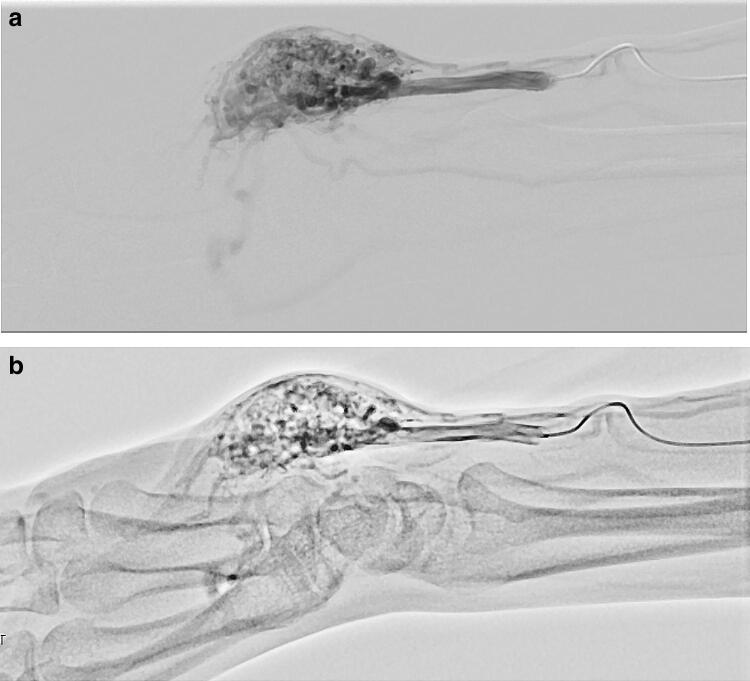
Rotational angiography of the right hand in a 43-year-old female with arteriovenous malformation of the right palm. While difficult to complete using DSA (**a**), DA with HF (**b**; Supplementary movie 1) facilitated the easy acquisition of rotational angiography to reveal a detailed three-dimensional relationship of such anatomically complicated vascular pathologies, particularly with reference to the carpal bones

HF has been clinically introduced in DA in conjunction with an FPD system to reduce the dynamic range; acquire DSA-like images without misregistration; and improve vessel delineation even in regions overlapped by radiopaque structures such as bones originally during angiography or IR of the lower extremities [7, 8]. In addition, when the operator visually traces arterial flow and timely scrolls the patient’s bed following a single injection of contrast media, use of HF in DA facilitates the easy acquisition of stepping angiography of the complete lower extremities much beyond the FPD coverage in a manner that is also difficult to realize with DSA and with better delineation of the wide-range vessels with less overlapped backgrounds than the original DA images (Fig. 1). This application can also make critical anatomical information regarding the target lesion for IR in relation to the background (e.g., bones) more visible [8]. To conduct DSA covering the complete lower extremities, multiple injections of contrast media and greater radiation exposure than that seen with DA are required. While difficult to complete with DSA, by involving HF in DA, we can also easily conduct rotational angiography to reveal the detailed three-dimensional relationship of complicated vascular anatomies and pathologies (e.g., arteriovenous malformation of the upper extremity) with less overlapped backgrounds than as seen without this function (Fig. 2).

HF is commercially available as not only “CLEAR LEG” but also “Dynamic Trace” from Canon Medical Systems (Otawara, Japan) and “SCORE RSM” from Shimadzu Medical Systems (Kyoto, Japan). In “CLEAR LEG,” the operator in real time can select 11 different dynamic ranges defined as DDO set values (in percentages) from 0% (i.e., the original DA) to 100% (10% intervals) in the DA images [7, 8]. The default DDO set value is 90% for angiography or IR of the lower extremities. In both “Dynamic Trace” (https://sg.medical.canon/products/angiography/more-information/angio_technology.html) and “SCORE RSM” (https://www.shimadzu-medical.eu/score-rsm), the dynamic range is optimized in advance and fixed for this purpose.

Using “CLEAR LEG,” we performed phantom experiments to optimize DDO set values for body IR because increased body thickness can affect the image quality [8]. Whereas the tube voltage should be increased, the dynamic range should be also widened to preserve image contrast between contrast media and background in body IR. As the DDO set value increases, image gradations are compressed, image contrast (e.g., density difference between the bone and soft tissues) is decreased, and halation artifacts are suppressed. Use of a DDO set value between 70 and 90% preserves the image contrast to some extent without significant halation artifacts arising from the lung field. The use of unsharp mask processing can generate bright band-like artifacts referred to as halo effects surrounding the vessel margins and improve the vessel conspicuity and delineation. As the DDO set value increases, this halo effect is also enhanced. Thus, we routinely use a DDO set value of 70–90% for body IR at our institution [8].

## Clinical advantages for body IR

### Avoidance of motion-related misregistration

The acquisition of DSA images of adequate quality and interpretability is one of the most important factors driving the successful performance of body IR procedures. Thus, the patient must hold still and limit their breath-taking during DSA image acquisition. As such, in patients who have difficulty doing so, misregistration in DSA is often problematic secondary to body and respiratory motion due to various reasons such as pain, restlessness, involuntary movement, dyspnea, impaired consciousness, mental retardation, and dementia. Misregistration can also occur related to motions uncontrollable by the patient such as bowel and ureter peristalsis and cardiac pulsation [4]. Conversely, even with the use of HF, misregistration does not typically occur in DA, thanks to no need of pre-contrast mask image acquisition.

Further, as the patient is sometimes required to hold his or her breath for a long time following a deep breath for DSA acquisition, that can increase the risk of catheter dislocation, leading to prolonged procedure time, degraded workflow, and increased radiation exposure in body IR. The use of HF in DA can offer real-time DSA-like images even under the patient’s free-breathing and reduce the risk of catheter dislocation.

### Identification of anatomical landmarks

**Fig. 3:**
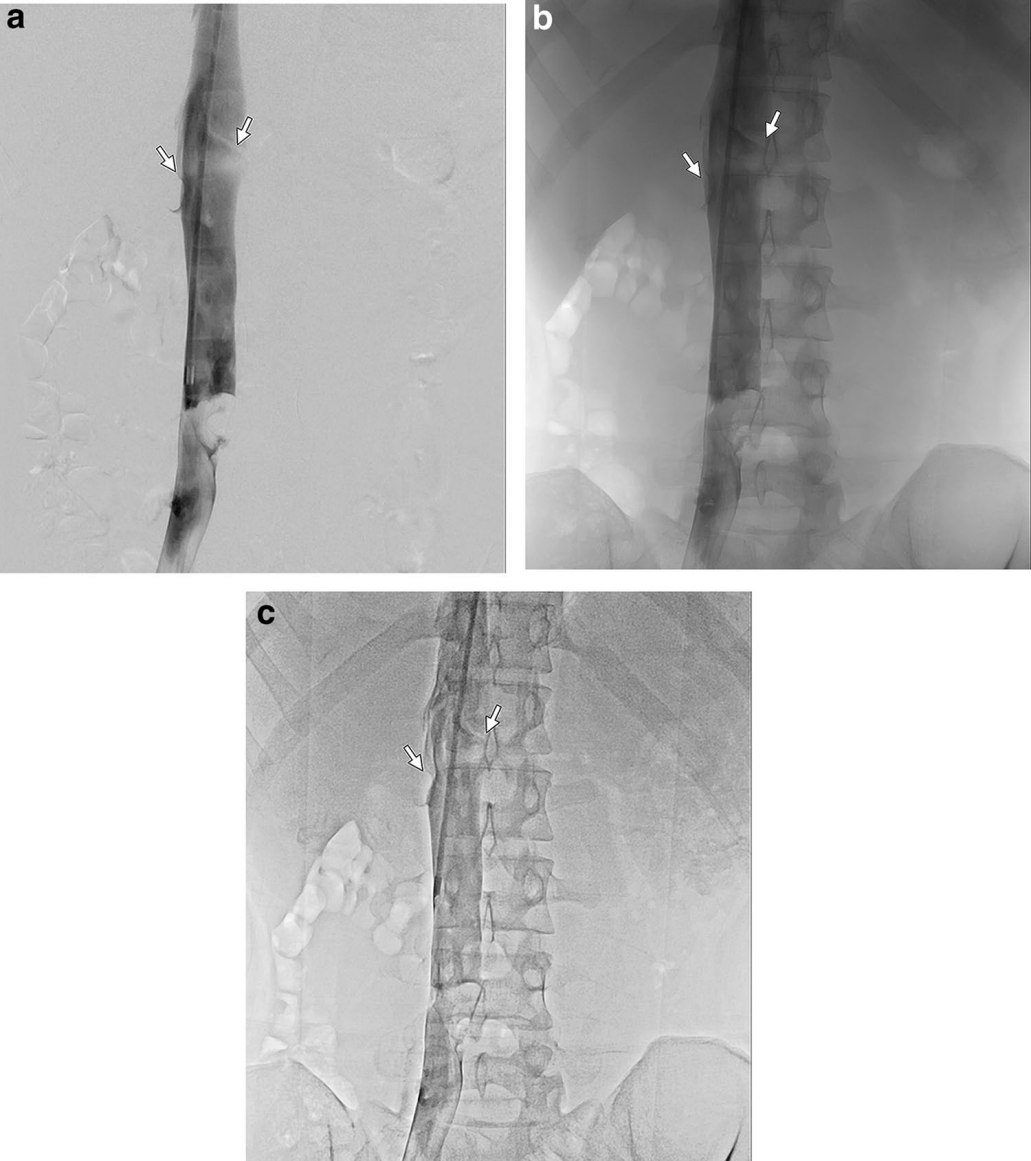
IVC filter placement in a 22-year-old male with deep venous thrombosis. DSA of the IVC (**a**) well delineates the confluence sites of the renal veins (arrows) without visualization of vertebrae. In contrast, DA (**b**, **c**) visualizes vertebrae as anatomical landmarks to determine the release site of the filter. Compared with DA without HF (**b**), DA with HF (**c**) better delineates these confluence sites with less of an overlapped background

The delineation of background radiopaque structures such as bones is eliminated in DSA but, sometimes, the presence of these structures is critical as anatomical landmarks for body IR procedures [10]. These structures are faintly visible even with high DDO set values in DA with HF. Specifically, during inferior vena cava (IVC) filter placement (Fig. 3), we often acquire DSA images of the cavography to identify confluence sites of the renal veins as filling defects of contrast media, using the vertebrae as anatomical landmarks to determine the release site of the filter by referring to DA images of the cavography and then actually release the filter under fluoroscopy. Use of HF can facilitate identification of the confluence sites of the renal veins in DA images of the cavography, offer fluoroscopy-like images with the vertebrae faintly visible—different from DSA—and improve the workflow of IR procedures.

### Reduction of radiation exposure

In principle, even with HF, acquisition of the mask image is not required in DA. When the quality and interpretability of DSA images are insufficient due to misregistration, additional radiation is often delivered for the re-acquisition of adequate DSA images. Thus, the use of HF in DA can ensure lower radiation exposure than DSA in combination with a better workflow of body IR procedures [8].

Using an FPD system from Siemens, the following default acquisition parameters are applied with an automatic exposure control for body IR: tube voltage, 70 kV; pulse width (= exposure time per image), 50 ms in DSA versus tube voltage, 70 kV; pulse width, up to 12.5 ms in DA with HF or “CLEAR LEG.” In DSA, the lowest tube voltage (i.e., 70 kV) is used to achieve the greatest image contrast between vessels or lesions opacified with contrast media and background and, thus, a longer pulse width (or a higher tube current–exposure time product [mAs]) is needed to preserve the signal–noise ratio. On the other hand, in “CLEAR LEG,” a higher tube voltage is needed to decrease the overlap generated by radiopaque structures and offer a homogeneous background like in DSA; while, the shorter pulse width is necessary to avoid motion-related misregistration. Thus, the mAs value is generally lower in “CLEAR LEG” than in DSA [8]. As per the results of our phantom experiments, the use of “CLEAR LEG” reduced radiation exposure by 50%–80%, while preserving image quality and interpretability relative to DSA. With HF in DA, if available, the operator should reduce radiation exposure to as low as reasonably achievable, particularly to help preserve fertility in young women who undergo uterine arterial embolization (UAE).

## Clinical applications for body IR

We, henceforth, present a series of clinical cases to demonstrate the aforementioned advantages of HF in DA for body IR.

## Transcatheter arterial embolization (TAE) for gastrointestinal hemorrhage

**Fig. 4:**
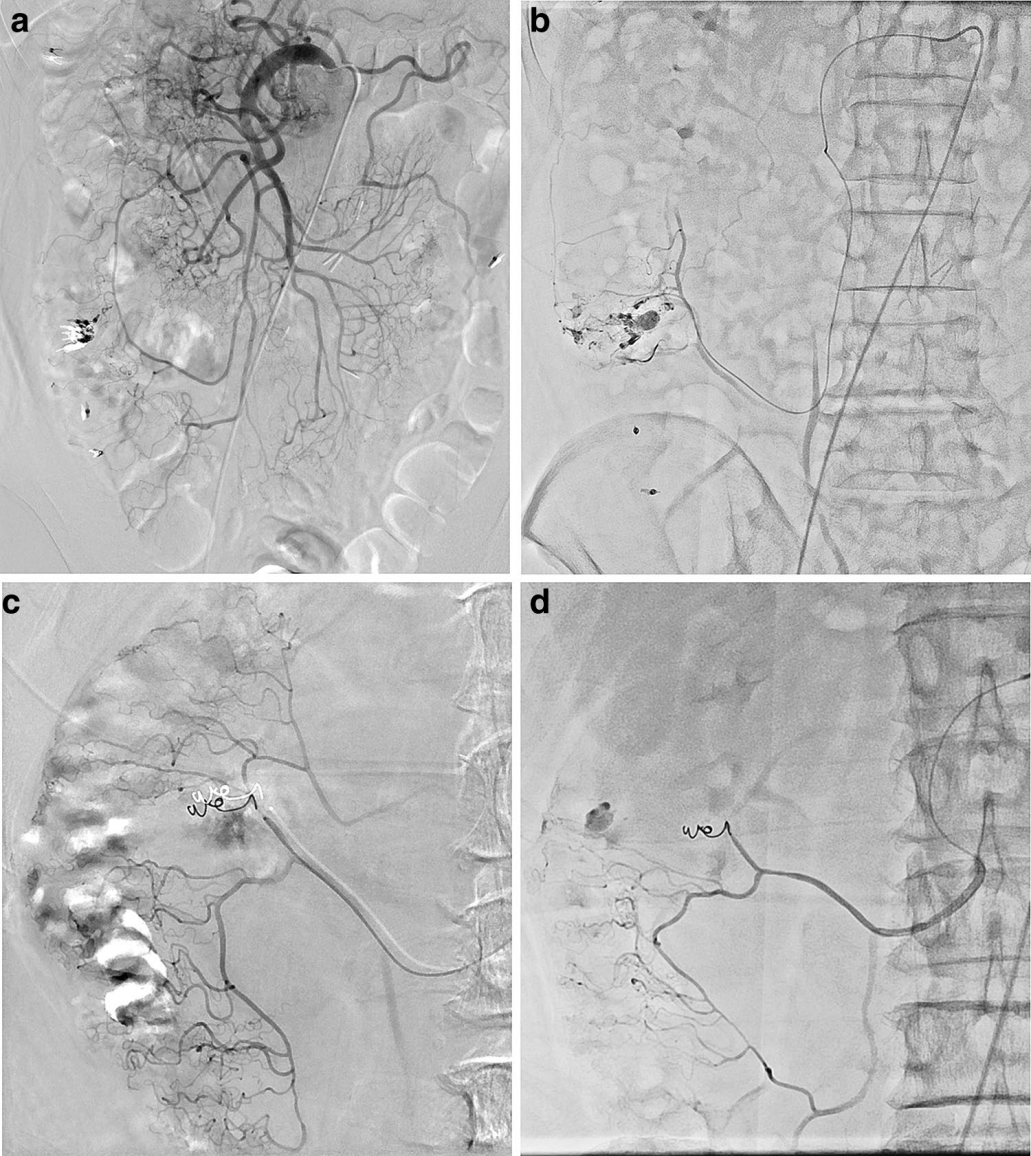
TAE for gastrointestinal hemorrhage in a 57-year-old male with hemorrhage from ascending colonic diverticula (**a**, **b**). Identification of extravasation of contrast media from the diverticulum is difficult, particularly in the presence of metallic clips, due to misregistration related to bowel peristalsis in DSA of the right colic artery (**a**) but is improved in the DA with HF (**b**) using an FPD system from Siemens. Separately, a 71-year-old male with hemorrhage from the ascending colonic diverticula is presented (**c**, **d**). As compared with DSA of the right colic artery (**c**), identification of extravasation of contrast media from the diverticulum is similarly improved in DA with HF (**d**) using the aforementioned system from Canon

In patients with colonic diverticular hemorrhage (Fig. 4) who underwent TAE at our institution using FPD systems from two different vendors [i.e., Siemens (Fig. 4a, b) and Canon (Fig. 4c, d)], we firstly acquired DSA via the superior mesenteric artery and its branches with a microcatheter to identify target vessels for TAE to achieve hemostasis [11]. Extravasation of contrast media from the diverticulum is, however, difficult to identify, particularly in the presence of metallic clips, because of misregistration related to bowel peristalsis. Thus, we also acquired DA images with HF and successfully identified the extravasation without misregistration using both FPD systems. This extravasation identification process is easier, clearer, and yields more definitive outcomes due to less background overlap than as seen in DA without HF. DA with HF sufficiently delineates small peripheral vessels like the vasa recta that are often critical target vessels in TAE for gastrointestinal hemorrhage.

**Fig. 5:**
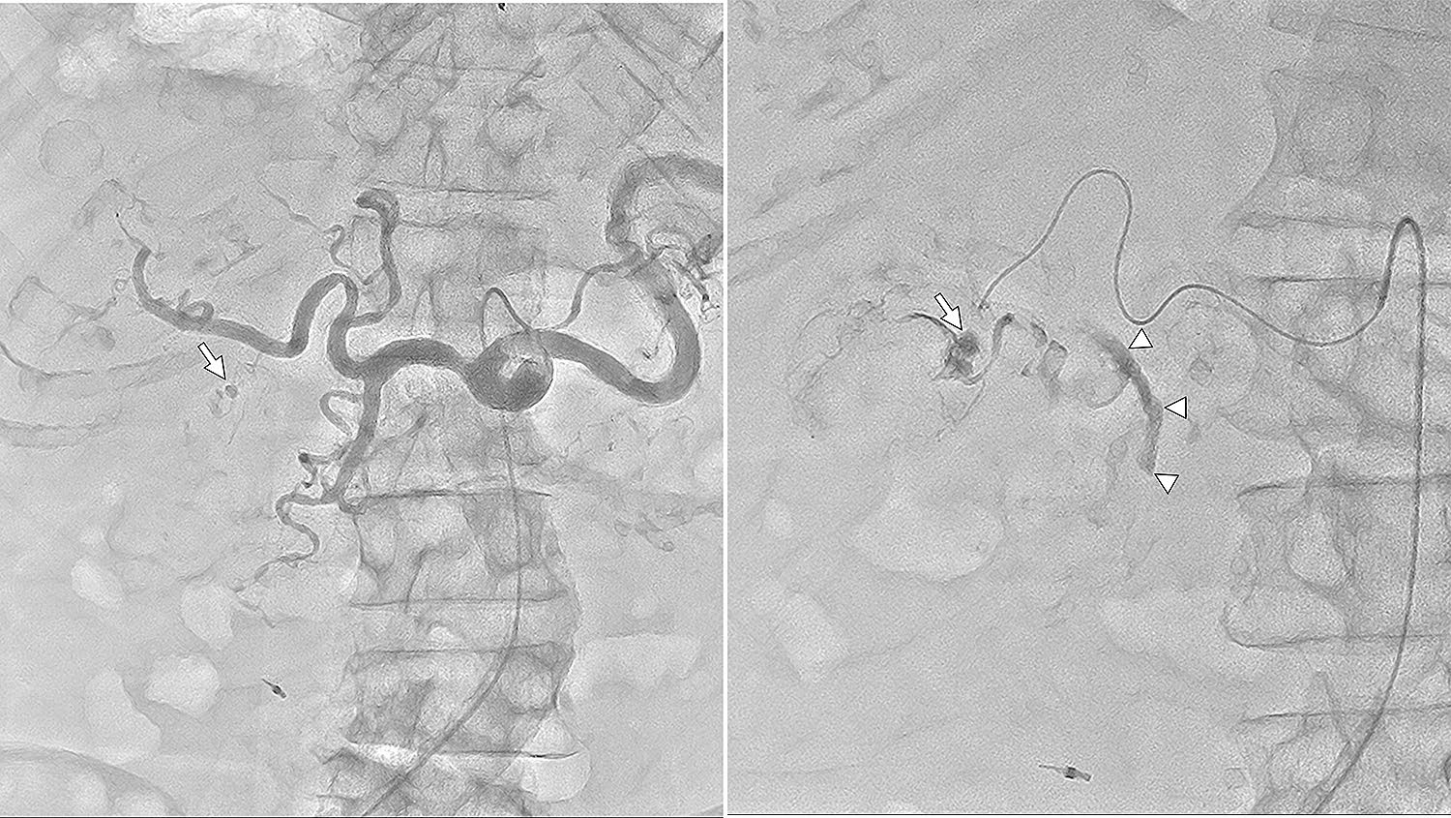
TAE for gastrointestinal hemorrhage in a 78-year-old male with hemobilia, hypovolemic shock, and unconsciousness. Free-breath DA with HF of the celiac artery (**a**) well delineates the cystic artery and its ruptured pseudoaneurysm (arrow). In that of the cystic artery (**b**), in addition to this excellent delineation of the pseudoaneurysm (arrow), extravasation of contrast media could be seen draining into the common bile duct (arrowheads) even in this patient who could not hold his breath using an FPD system from Canon

In a patient with endoscopically confirmed hemobilia resulting from a ruptured pseudoaneurysm of the cystic artery (Fig. 5) who presented with hypovolemic shock and unconsciousness and could not hold his breath, we firstly acquired DSA via the celiac artery (CA) to identify target lesions for TAE. Such lesions are, however, difficult to identify because of motion artifacts and misregistration related to his insufficient breath-holding. Instead, we acquired DA with HF via the CA and proper hepatic artery with a microcatheter and successfully identified the pseudoaneurysm from which extravasation of contrast media was drained into the biliary tract in these DSA-like images. Different from DSA, use of DA with HF does not require patient’s breath-holding to avoid misregistration and is ideal among critically ill patients who cannot hold their breath.

## Transcatheter arterial chemoembolization (TACE) for hepatocellular carcinoma (HCC)

**Fig. 6:**
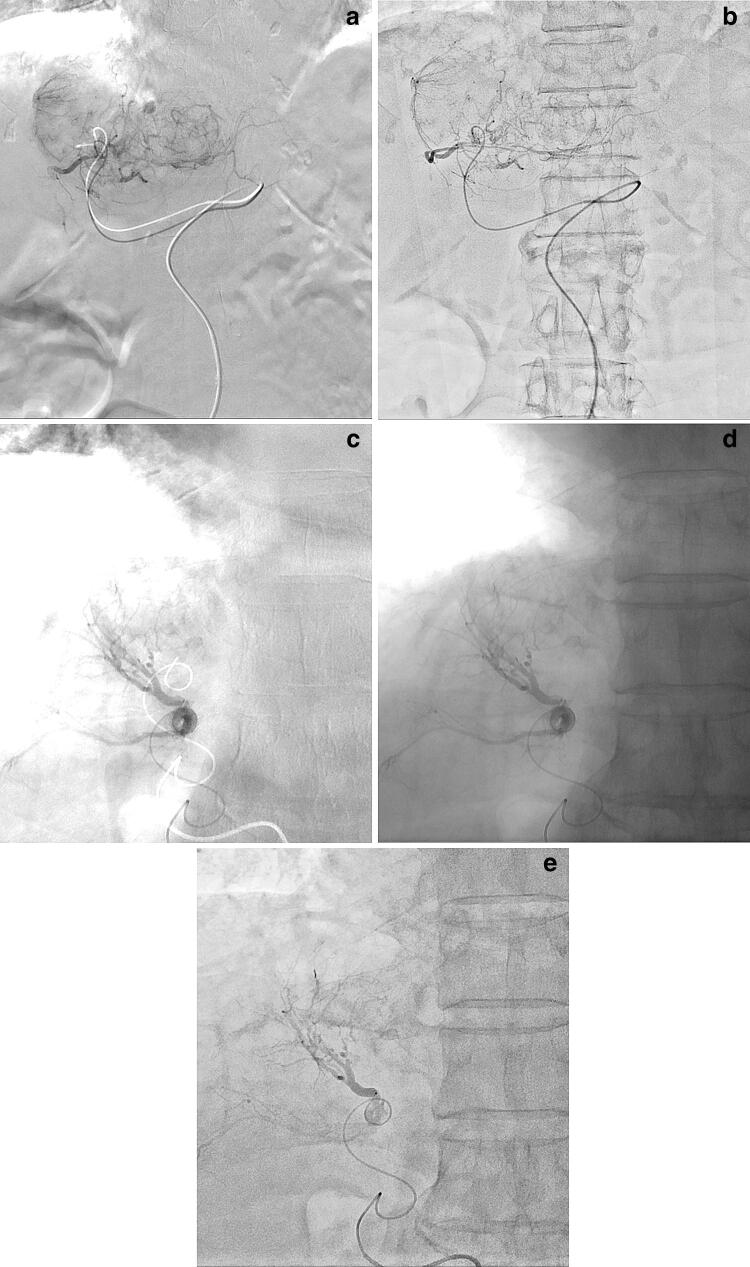
TACE for HCC in an 85-year-old female with subdiaphragmatic HCC (**a**, **b**). In this patient who could not sufficiently hold her breath, delineation of the entire tumor stain was noted as severely degraded due to respiration-related misregistration and halation artifacts from the lung field in DSA of the left hepatic artery (**a**), but was improved in the free-breath DA with HF (**b**). Separately, a 64-year-old male with recurrent HCC (**c**–**e**). In this patient who could not sufficiently hold his breath, respiration-related misregistration and halation artifacts from the lung field are severe in DSA (**c**). The misregistration is eliminated but the halation artifacts still remain in DA without HF (**d**). On the other hand, both the misregistration and halation artifacts are successfully eliminated in DA with HF (**e**)

In patients who undergo TACE for HCC, the accurate identification of feeding arteries and tumor stains is critical to complete often using DSA [12], which can better delineate even fine feeding arteries and tumor stains adjacent to lipiodol accumulation than DA, even with HF. Potential feeding arteries to HCC other than the hepatic arteries include the inferior phrenic artery, particularly for subdiaphragmatic HCC. Quality and interpretability of DSA images in the subdiaphragmatic region are susceptible not only to cardiac pulsation but also to respiration-related diaphragmatic motion, which can cause motion-related misregistration and halation artifacts from the lung field (Fig. 6). The identification of feeding arteries and tumor stains of subdiaphragmatic HCC can be easily degraded in DSA, particularly in those patients who cannot sufficiently hold their breath during imaging. Among these patients, free-breath DA with HF is a beneficial option for overcoming the shortcomings of DSA, also in the presence of lipiodol accumulation, because it can offer DSA-like images unsusceptible to respiratory motion (Fig. 6).

DA with HF can offer lower radiation exposure and better procedural workflow than DSA because it does not require the acquisition of pre-contrast mask images or breath-holding by the patient. In TACE for HCC, DSA images adequately acquired in cooperation with the patient are critical for the detailed assessment of feeding arteries and tumor stains to help drive treatment strategy selection. Otherwise, DSA is often conducted also for simply confirming whether the catheter placement is adequate or not, whether the embolization effect is satisfactory or not, and so forth. DSA can be replaced by DA with HF for these purposes and we, thus, successfully can reduce radiation exposure and improve the procedural workflow in TACE for HCC at our institution. DA with HF, if available, may be the first choice also in emergency TAE or TACE for critically ill patients with HCC rupture who cannot hold their breath.

## TAE for renal angiomyolipoma (AML) in tuberous sclerosis complex (TSC)

**Fig. 7:**
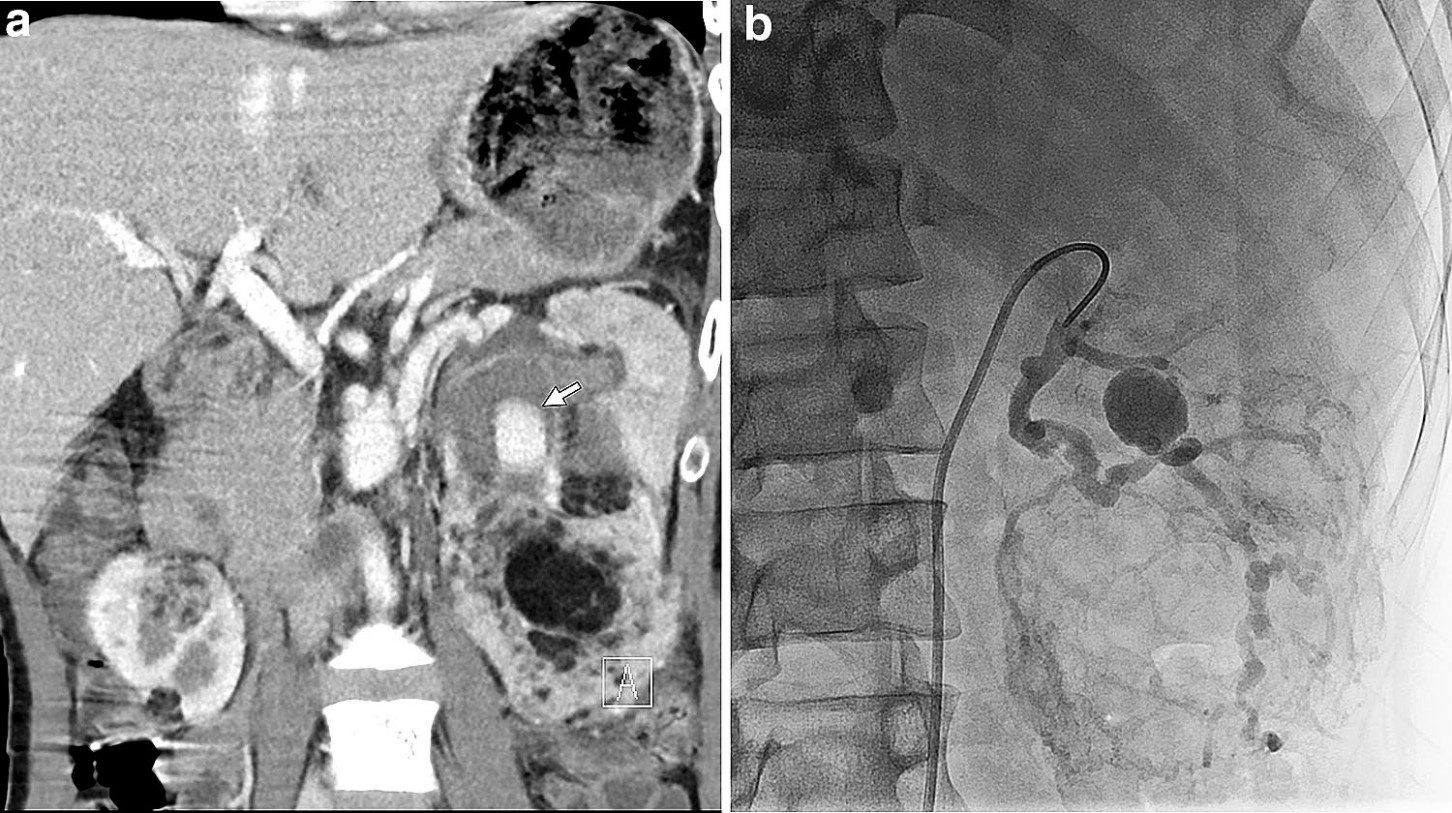
TAE for renal AML in TSC in a 29-year-old male with TSC and mental retardation. A multiplanar reconstruction coronal image of post-contrast abdominal computed tomography (**a**) shows multiple AMLs in both the kidneys and an aneurysm (arrow) within an AML in the left kidney. Note that a motion artifact is shown due to patient body motion caused by mental retardation. In this patient who could not hold his breath under sedation, we did not plan to acquire DSA but only acquired free-breath DA of the left renal artery with HF (**b**), where no respiration-related misregistration is shown. Background overlap is a little marked in this image acquired with the DDO set value of 60%, which is lower than the routine value (i.e., 70–90%)

TSC is an autosomal dominant inherited disorder characterized by generalized involvement and variable manifestations occurring at different times throughout the individual’s lifespan. The presence of an aneurysm within the AML correlates with the risk of hemorrhage. In Japan, growing asymptomatic AMLs larger than 4 cm in diameter and/or with an aneurysm larger than 5 mm in diameter are recommended to undergo prophylactic TAE [13–16]. Emergency TAE is also indicated in patients with ruptured AMLs and/or aneurysms. In patients with TSC-associated neuropsychiatric disorder [17–21], sedation is often required to prevent body movement during these TAE procedures, and DA with HF is a useful alternative to DSA in this context for offering DSA-like images without respiration-related misregistration (Fig. 7). As a result, the use of DA with HF can reduce radiation exposure, improve the workflow of TAE procedures, and decrease the procedure time in these cases.

## Adrenal venous sampling (AVS)

Unilateral primary aldosteronism is the most common surgically curable form of endocrine hypertension and is usually differentiated from bilateral forms by AVS. According to a review article, the success rate when cannulating both the adrenal veins in a large cohort was 74%; with experience, the success rate increased to 96% [22–26].

**Fig. 8:**
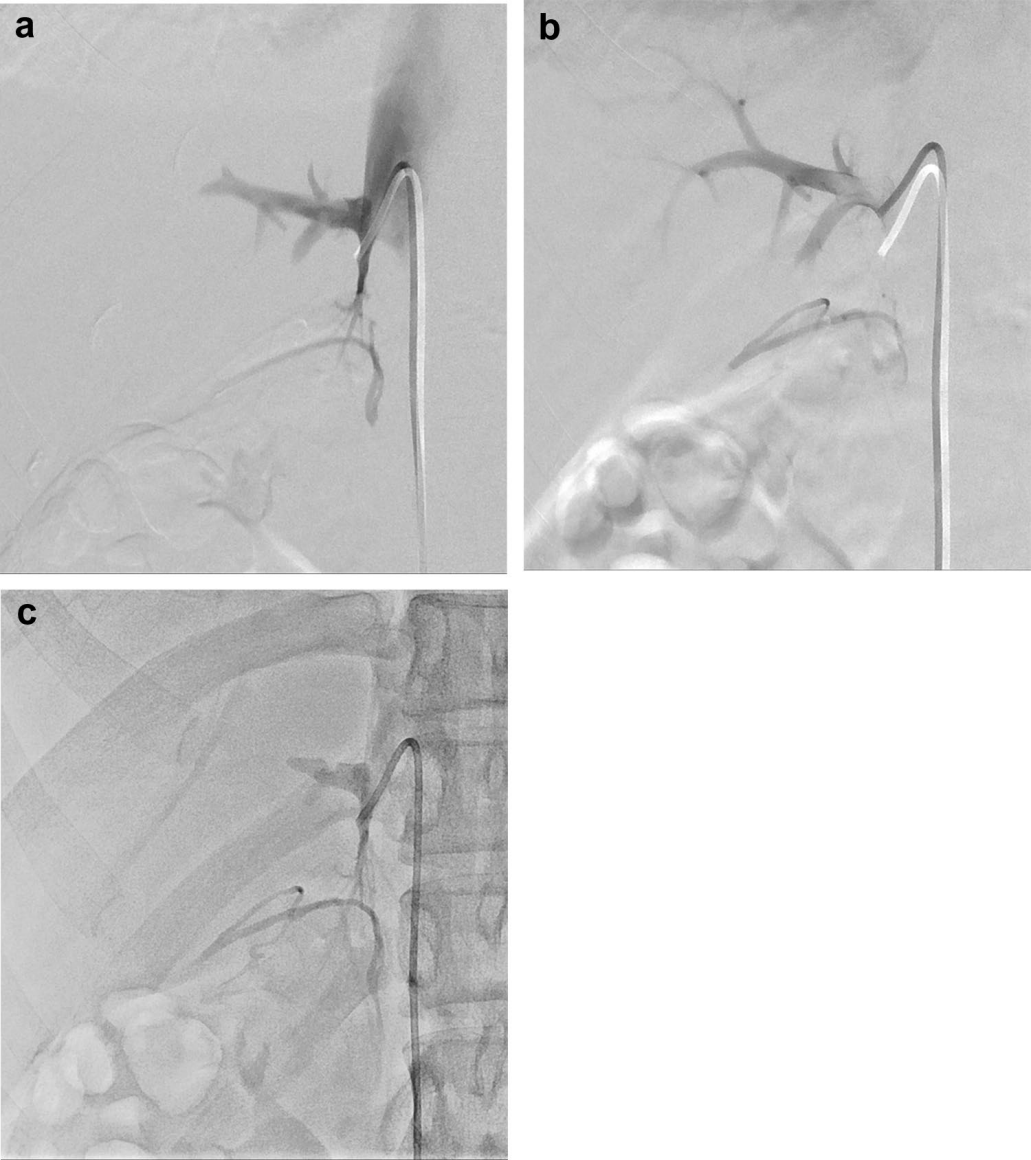
AVS in a 23-year-old female with primary aldosteronism. Successful catheterization into the right adrenal vein was confirmed in DSA of the right adrenal vein during her breath-holding (**a**) but then catheter dislocation from the right adrenal vein into the accessory hepatic vein was noted in the DSA just after her breath-holding (**b**; Supplementary movie 2). Successful re-catheterization into the right adrenal vein was confirmed in the free-breath DA with HF (**c**)

Catheterization into the right adrenal vein is the primary difficult task because of anatomical factors, which include the small diameter, short length, and direct drainage into the IVC. Catheter dislocation can also more easily occur from the right adrenal vein, particularly just before or after the patient holds their breath for DSA acquisition (Fig. 8). Insufficient breath-holding by the patient can promote respiration-related misregistration, poor quality and interpretability in DSA images of adrenal venography, and poor confirmation of catheterization into the adrenal veins. Free-breath DA with HF can overcome these shortcomings of DSA (Fig. 8). In addition, using DA with HF instead of DSA, the vertebrae that are faintly visible can be utilized as anatomical landmarks for re-catheterization into the right adrenal vein on fluoroscopy-like images following catheter dislocation. Thus, the use of DA with HF can improve the success rate of bilateral AVS, which is approximately 100% at our institution.

## UAE for postpartum hemorrhage

**Fig. 9:**
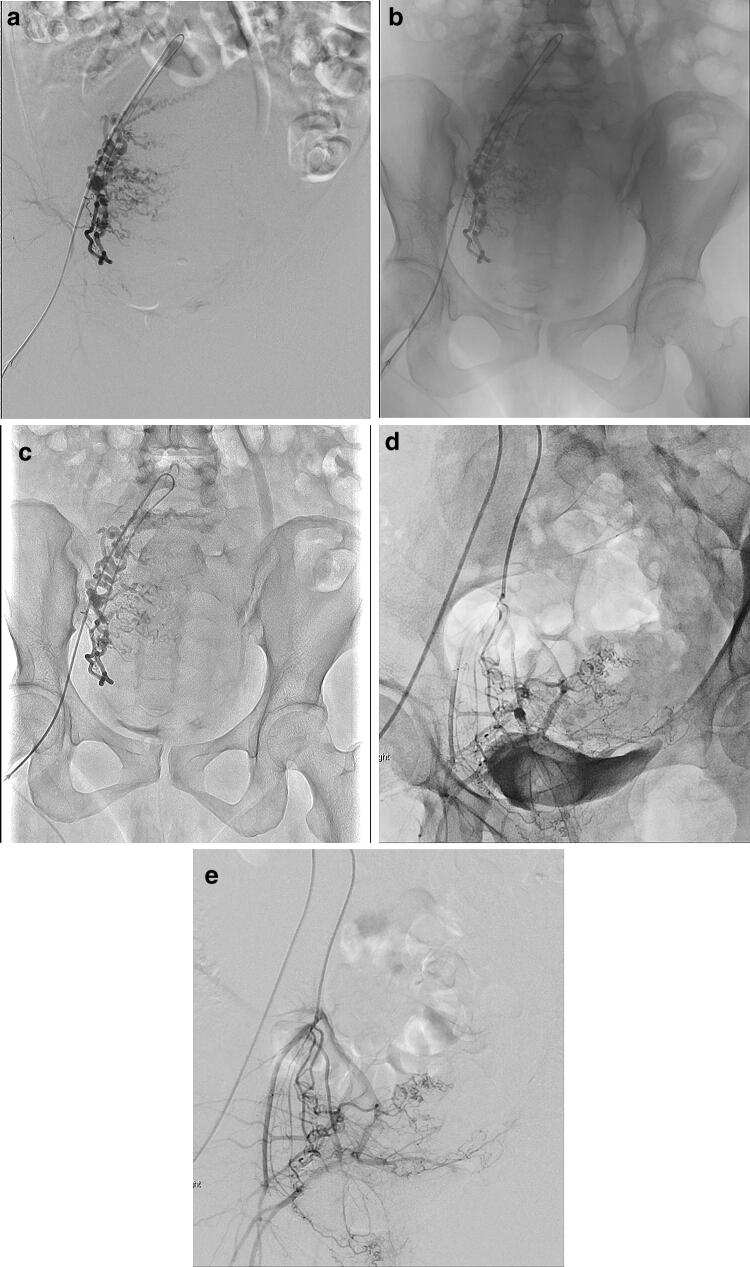
UAE for postpartum hemorrhage. A 39-year-old female with retained placenta (**a**–**c**). In this patient who could not sufficiently hold her breath, respiration- and peristalsis-related misregistration of bowel gas was observed as severe in DSA of the right uterine artery (**a**) but eliminated in the free-breath DA (**b**, **c**). Compared with DA without HF (**b**), DA with HF (**c**) improves image contrast of the uterine artery to an overlapped background. Separately, a 32-year-old female with retained placenta (**d**, **e**) is presented. Contrast medium pooled in the urinary bladder interferes with delineation of the internal iliac arteries and their branches in DA of the right uterine artery even with HF (**d**), different from DSA (**e**)

As emergency IR, UAE is increasingly indicated to avoid hysterectomy in patients with critical bleeding in obstetrics (e.g., postpartum hemorrhage) with the clinical success rate of approximately 79–100% [27–30]. In general, latter emergency IR procedures are more complicated and require longer procedure times and increased radiation exposure. In patients undergoing these IR procedures, radiation exposure should be reduced to as low as reasonably achievable, particularly to help preserve their fertility. Because DA with HF can offer lower radiation exposure than DSA, the use of DA with HF, if available, should be considered as an alternative to DSA, particularly for simple confirmation of the catheter location or embolization effect. This replacement of DSA by DA with HF can not only reduce misregistration related to bowel and ureter peristalsis and/or insufficient breath-holding but also improve the IR workflow and reduce the procedure time (Fig. 9).

## Bronchial artery embolization (BAE) for hemoptysis

**Fig. 10:**
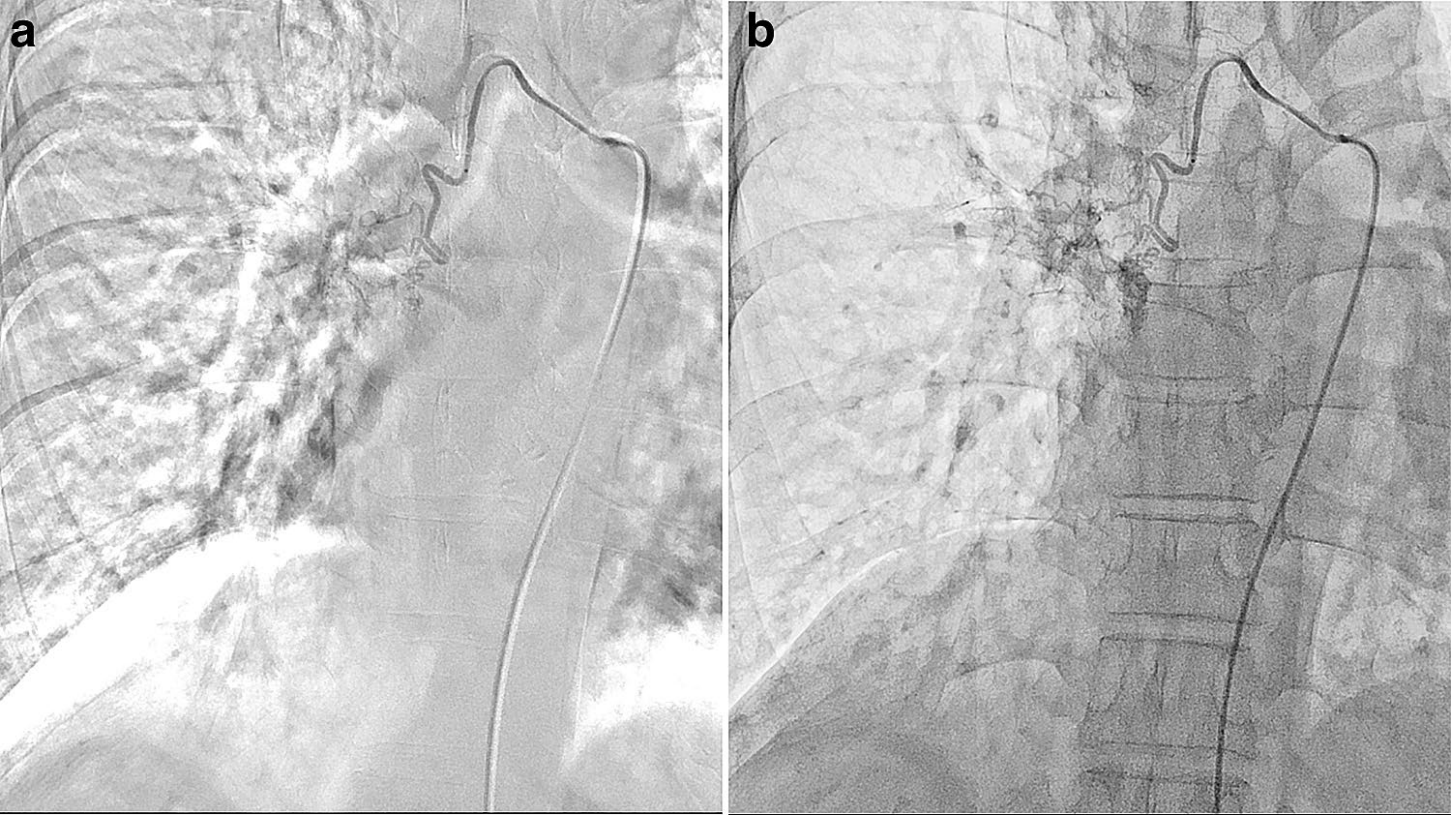
Bronchial artery embolization for hemoptysis in a 50-year-old female with massive hemoptysis and severe respiratory insufficiency. In this patient who could not hold her breath, respiration-related misregistration and halation artifacts from the lung field appeared as severe in DSA of the right bronchial artery (**a**) but were eliminated in the free-breath DA with HF (**b**)

BAE is sometimes indicated as an emergency IR procedure in patients with hemoptysis who have respiratory insufficiency and/or who are critically ill [31–34]. Potential target vessels in BAE for hemoptysis include bronchial arteries and non-bronchial systemic arteries such as the internal thoracic, lateral thoracic, dorsal thoracic, and intercostal arteries. Such vessels should be swiftly and adequately visualized to determine the target vessels for the BAE. This visualization by use of DSA, however, can be easily degraded not only by halation artifacts from the lung field and/or misregistration related to cardiac pulsation but also by respiration-related misregistration, which is particularly problematic in patients who cannot hold their breath. Free-breath DA with HF is a useful alternative to DSA for eliminating these artifacts in BAE. In a patient with massive hemoptysis and severe respiratory insufficiency who had concurrent diseases such as Down syndrome, ventricular septal defect, pulmonary hypertension, and Eisenmenger’s syndrome (Fig. 10), all of the aforementioned artifacts were markedly noted in DSA but eliminated in free-breath DA with HF; thus, we successfully performed BAE to achieve hemostasis at our institution.

## IR in other regions

Other than body IR, in cardiac IR where DSA is not routinely acquired, the DA with HF is expected to improve delineation of fine vessels distributed over dense areas such as the diaphragm and vertebra [7]. Although we have never applied the DA with HF in head and neck IR, this technique may be less useful in this region because misregistration related to bowel peristalsis, cardiac pulsation, or respiratory motion is not problematic in DSA. Otherwise, however, the DA with HF may be similarly advantageous also in head and neck IR.

## Limitations for body IR

Although DA with HF is clinically useful in a wide variety of body IR procedures and, if available, should be considered for replacing DSA depending on the acquisition purposes, it has several limitations as follows.

First, the availability of HF is still limited in pairing with FPD systems from some vendors. Second, the image contrast between vessels or lesions opacified with contrast media and background is increased from DA without HF to DA with HF to DSA. Particularly, the delineation of small and peripheral vessels and subtle tumor stains such as small HCCs in DA with HF is superior to that in DA without HF but inferior to that in DSA. When contrast media are too diluted with heparinized saline solution, vessel delineation is more easily degraded in DA with HF than DSA. The use of DA with HF may require a greater iodine load and impart a higher risk in patients with renal insufficiency as compared with DSA. Third, the delineation of vessels or lesions opacified with contrast media adjacent to highly radiopaque materials such as metallic implants, dense calcification, and lipiodol accumulation is worse in DA, even with HF, than in DSA, which can subtract these radiopaque materials. Specifically, contrast media pooled in the urinary bladder can easily interfere with delineation of the internal iliac arteries and their branches (Fig. 9c, d). Thus, a balloon catheter should be preoperatively placed in the urinary bladder in UAE, TAE for pelvic fractures, and so forth. Finally, radiation exposure in DA with HF is much lower than in DSA but is a little higher than in DA without HF. Further technological advances are expected to reduce radiation exposure in these FPD systems.

## Conclusions

DA with HF as a good alternative to DSA is clinically advantageous in body IR for offering DSA-like images and simultaneously reducing various motion artifacts and misregistrations caused by patient body motion, poor breath-holding, bowel and ureter peristalsis, and cardiac pulsation as well as halation artifacts stemming from the lung field. Free-breath DA with HF can improve body IR workflow and decrease the procedure time by decreasing the risk of catheter dislocation and using background structures as anatomical landmarks while reducing radiation exposure when compared with DSA. HF should be more widely and effectively utilized for adequate purposes in body IR based on sufficient knowledge and understanding of the principles and clinical usefulness and limitations of this technology.

This article was presented in part at the 104th annual meeting of the Radiological Society of North America in Chicago, 2018. It received a Certification of Merit as an educational exhibit.

## Electronic supplementary material

Below is the link to the electronic supplementary material.

Supplementary material 1 (MPG 1040 kb)

Supplementary material 2 (MPG 368 kb)
